# Observed Clinical, Laboratory, and Echocardiographic Parameters in Takotsubo Syndrome Patients with Mortality and Decreased Ejection Fraction During Initial Hospital Admission

**DOI:** 10.51894/001c.6941

**Published:** 2018-09-26

**Authors:** Andrew Hinojos, Thomas E. Vanhecke, Stephen Manning

**Affiliations:** 1 Genesys Heart Institute, PGY-5 Cardiology Fellow, Grand Blanc, MI; 2 Genesys Heart Institute Attending Cardiologist, Grand Blanc, MI; 3 Genesys Regional Medical Center/Ascension Health PGY-1 Internal Medicine Resident, Grand Blanc, MI

**Keywords:** heart failure, cardiomyopathy, acute coronary syndrome, takotsubo syndrome

## Abstract

**CONTEXT:**

Approximately 1-2% of patients with suspected acute coronary syndrome also develop Takotsubo syndrome (TTS). This syndrome is characterized by transient systolic dysfunction of the apical and/or mid segments of the left ventricle that mimics myocardial infarction in the absence of obstructive coronary artery disease. Up to 21.8% of TTS patients develop serious complications, including death. Currently, there is no consensus on management of these patients and their complications. Thus, identifying TTS patients at higher risk for complications becomes valuable in managing their hospital course. The aim of this study was to examine the predictive significance of laboratory, echocardiographic, and clinical parameters on in-hospital mortality in a sample subgroup of TTS patients. Secondary analyses were performed on patients with reduced (i.e., <35%) ejection fractions.

**METHODS:**

This retrospective study at a community hospital identified patients from October 1, 2009 to August 31, 2015 who presented with ACS and underwent cardiac catheterization. Patients were diagnosed with TTS by features of cardiomyopathy on cardiac catheterization or echocardiogram.

**RESULTS:**

The authors analyzed data from a total of 177 eligible patients identified with TTS. The in-hospital mortality rate was 5.65%. Compared to the non-mortality subgroup, patients who suffered in-hospital mortality had significantly lower diastolic blood pressure on admission (p < 0.050), lower hemoglobin levels (p < 0.001), lower sodium (p = 0.020), higher blood urea nitrogen (p = 0.009), lower glomerular filtration rate (p = 0.016), and lower albumin levels (p < 0.001). Cox regression analyses demonstrated admission hemoglobin was significant, yielding a mortality hazard ratio of 0.760 (95% CI of 0.594-0.972, p = 0.029).

**CONCLUSIONS:**

Patients who present with TTS and hypotension, anemia, low albumin levels, elevated lactic acid and renal dysfunction were associated with higher rates of in-hospital mortality in this study’s sample population. Further, admission hemoglobin had the strongest association with death. Every unit decrease in hemoglobin increased mortality risk by 24%.

## INTRODUCTION

Takotsubo (or “Tako-Tsubo”) syndrome (TTS) is characterized by transient systolic dysfunction of the apical and/or mid segments of the left heart ventricle mimicking myocardial infarction in the absence of obstructive coronary artery disease (CAD).^1,2^ Approximately 1-2% of patients with suspected acute coronary syndrome (ACS) have been found to have TTS after cardiac catheterization.^3^ Several studies have shown that TTS is not a benign or transient syndrome, with complications in up to 21.8% of patients.^4–6^

Most common complications occur in the acute setting and include heart failure, tachyarrhythmias, mitral regurgitation, left ventricular (LV) outflow tract obstruction, and cardiogenic shock.^2,7–9^ Mortality rates have ranged from 1.1% to 10.2%.^3,5,6,8–12^ Although the pathogenesis of TTS is not completely understood, experts have concluded this syndrome is most likely due to a surge of catecholamines causing vascular dysfunction and direct myocardial stunning (i.e., reversible reduction of function of heart contraction after reperfusion).^2,13–15^

Clinicians’ efforts to identify TTS patients at higher risk for complications and mortality are imperative, as traditional therapies for heart failure and cardiogenic shock have been limited and shown mixed results.^16^ Use of beta blockers to attenuate the cathecholamine surge does not appear to improve mortality, complication rates, or prevented recurrence.^5,17,18^ Additionally, information regarding use of angiotensin converting enzyme inhibitors (ACEI) or aldosterone receptor blockers (ARB) is limited with potential benefits demonstrated in one retrospective study.^18^

In the severely ill TTS patient, management presents unique complications such as dynamic left ventricular outflow tract (LVOT) obstruction. Use of inotropes is generally regarded as a contraindication in TTS as they can worsen the dynamic LVOT obstruction.^2,19^ Previous case reports have also suggested poor outcomes with use of beta agonists and vasopressors.^19,20^ Thus, it remains critical to identify those TTS patients at greater risk for complications since there is still little conclusive evidence for treatment available.^2,5^

### Purpose of Study

The specific aim of the study was to examine the significance of key laboratory, clinical, and echocardiographic parameters observed in a sample subgroup of TTS patients whose hospital course was complicated and/or may have experienced in-hospital mortality.

## METHODS

This study was a retrospective chart review approved by the local institutional review board in January, 2017 and conducted at a community-based hospital in Grand Blanc, Michigan. Patient data were obtained through electronic health records pulled by the first (AH) and third (SM) authors using ICD-9 codes 410.9 (myocardial infarction) and 429.83 (Takotsubo Syndrome) from October 1, 2009 to August 31, 2015.^21^ The convenience sample included patients ages 18 and older who had presented as an acute myocardial infarction and underwent cardiac catheterization during their hospital stays.

Charts were reviewed independently by two separate cardiologists, and patients’ TTS diagnosis was based on the Mayo Clinic Criteria and the 2016 European Society consensus statement.^1,2^ TTS was diagnosed in patients who had presented with ACS and demonstrated regional wall motion abnormalities without culprit atherosclerotic CAD (i.e., lesion(s) involved in an acute myocardial infarction).^22,23^

Each sample patient had received a cardiac catheterization to rule out acute plaque rupture, thrombus formation, coronary dissection or other pathological conditions that would explain observed patterns of temporary LV dysfunction. Patients were excluded from analyses if they had not received a cardiac catheterization, were referred for a coronary artery bypass graft, had failed a percutaneous coronary intervention, or showed no evidence of cardiomyopathy on either their cardiac catheterization or echocardiogram.

Ultimately, a total of 177 TTS patients were included for study analysis. Patients’ charts were also reviewed to extract sociodemographic information, clinical data, transthoracic echocardiogram, and cardiac catheterization results. The authors used the Killip classification method to stratify sample patients’ by clinical signs of heart failure.^24^

In this study, primary TTS was defined as patients whose primary reason for presentation was due to TTS cardiomyopathy. Secondary TTS was defined as patients who developed TTS secondary to another critical physical illness during admission.^2,3^

Patients’ degree of CAD was quantified by report with no abnormal coronary arteries assigned a value of 0, minimal or mild CAD assigned a value of 1, moderate CAD or any lesion >50% stenosis given a value of 2, severe CAD or any lesion >90% or three or more lesions >70% assigned a value of 3.^22,23^

Echocardiogram reports were reviewed for instances of any mitral regurgitation, aortic stenosis, and aortic regurgitation. Degree of valvular dysfunction was assigned a value: none or trace was given a value of 0, mild was assigned a value of 1, moderate a value of 2, and severe a value of 3.^25^

Data were analyzed by statisticians in the authors’ clinical research department using SPSS version 23.^26^ Dependent variables measured on a continuous scale were analyzed for significance using the independent samples t-test assuming normal distribution. Categorical data were analyzed for significance using the chi-square test for independence. Most of the results reported in this paper are presented as continuous variables expressed as a mean ± standard deviation or counts with population as appropriate.

The authors’ primary analytic outcome was to identify significant predictors of in-hospital mortality. Secondary analytic outcomes included predictors of ejection fraction (EF) < 35% and to identify medical therapy used during patients’ hospital course to examine associations with outcomes.^27^

## RESULTS

A total of N = 177 sample patients who met TTS criteria were identified. There were 10 (5.65%) instances of patient mortality. Overall, 79 (44.7%) patients demonstrated the typical apical ballooning type pattern. Primary TTS represented 155 (87.6%) patients while secondary TTS represented 22 (12.4%) patients. Mortality occurred in nine (5.8%) primary TTS patients and one (4.7%) secondary TTS patient. The most common causes of secondary TTS included gastrointestinal bleed, sepsis, and post-surgical causes. Etiologies of patient death were determined to be from ventricular tachycardia/fibrillation arrest in 40%, pulseless electrical activity arrest 50%, and undetermined in 10%.

Of the 45 (25.4%) of TTS patients identified as presenting with a ST segment elevation myocardial infarction, 18 (40%) were in the mortality subgroup versus 11 (24.5%) in the non-mortality subgroup. Medications used during the hospital course were analyzed between patients in the mortality and non-mortality subgroups. Overall, 79 (44.6%) of total sample patients had been placed on atorvastatin, 88 (49.7%) patients were placed on metoprolol tartrate, 60 (33.9%) of patients were placed on carvedilol, and 65 (36.7%) of patients were placed on Lisinopril.

Of the total sample, 33 (18.6%) patients showed moderate-to-severe mitral regurgitation (MR) on their echocardiogram. The mortality rate was 9.1% for patients with moderate-to-severe MR versus 4.9% in patients with mild or less MR. Patients with moderate-to-severe MR were significantly older (p = 0.016), had a significantly lower EF (p = 0.008), lower admission hemoglobin (p = 0.001), higher admission BUN (p = 0.004), and higher admission creatinine (p = 0.011). There were no other significant differences identified between subgroups, although left ventricular end-diastolic pressure (LVEDP) levels were somewhat higher in patients with moderate-to-severe MR, although this difference was not statistically significant (p = 0.071).

### Mortality versus Non-Mortality Subgroups

Baseline data between the mortality and non-mortality subgroups are represented in Table 1. In the mortality subgroup, 50% of the population was male versus 22.8% in the non-mortality subgroup (p = 0.064). Apical ballooning was found in 30% of patients in the mortality subgroup versus 43.7% in the non-mortality subgroup (p = 0.496).

**Table:** Table 1 Demographics and Clinical Characteristics of Study Population Stratified By Mortality versus Non-Mortality Subgroups

	**Mortality** **(n=10)**	**Non-Mortality** **(n=167)**	**p-value**
	**Mean (±SD)**	**Mean (±SD)**	
Age	66.4(±16.38)	63.73(±13.41)	0.547
BMI	31.99 (±9.11)	29.29 (±7.63)	0.285
BSA	2.06(±0.34)	1.92(±0.32)	0.148
LOS	7.8 (±8.12)	5.13(±6.75)	0.230
Day of Cardiac Catheterization	1.8(±1.03)	2.3(±2.2)	0.476
Clinical History **	**Frequency**	**Frequency**	**p-value**
Male	50.00%	22.80%	0.064
Female	50.00%	77.20%	0.064
Previous PCI	10.00%	9.60%	0.999
Previous CABG	40.00%	2.40%	**0.001**
PAD	10.00%	1.80%	0.209
CVA	10.00%	6.00%	0.483
Family History of CAD	10.00%	11.40%	0.999
Diabetes	40.00%	20.40%	0.225
HTN	70.00%	65.90%	0.999
COPD	30.00%	16.80%	0.383
OSA	20.00%	7.20%	0.181
Smoker	60.00%	57.50%	0.999
History of cancer	50.00%	13.80%	**0.010**
History of heart failure	20.00%	8.40%	0.225
ESRD	40.00%	13.20%	**0.042**
Depression	20.00%	32.30%	0.507

* Significant p values appear in **bold** font.

** Body mass index (BMI), Body surface area (BSA), Length of stay (LOS), Percutaneous coronary intervention (PCI), Coronary artery bypass graft (CABG), Peripheral artery disease (PAD), Cerebrovascular accident (CVA), Coronary artery disease (CAD), Hypertension (HTN), Chronic obstructive pulmonary disease (COPD), Obstructive sleep apnea (OSA), End-stage renal disease (ESRD).

Table 2 demonstrates the clinical, laboratory, and echocardiographic parameters obtained between the mortality and non-mortality subgroups. As shown in this table, patients who died during hospitalization had significantly lower mean admission hemoglobin levels (p < 0.001), lower admission hematocrit levels (p= 0.001), lower admission sodium levels (p = 0.02), higher admission BUN levels (p = 0.009), lower admission glomerular filtration rate levels (p = 0.016), higher admission lactic acid (p < 0.001), and lower admission albumin levels (p <0.001).

**Table:** Table 2 Cardiac Biomarkers, Admission Vitals and Laboratory Data for the TTS Subgroups

**Cardiac Biomarkers**	**Mortality** **(n=10)**	**Non-Mortality** **(n=167)**	**p-value**
	**Mean (±SD)**	**Mean (±SD)**	
Average Troponin (ng/ml)	0.43(±0.44)	2.28(±6.74)	0.440
Peak Troponin (ng/ml)	0.45(±0.44)	3.5(±11.05)	0.410
Admission Troponin (ng/ml)	0.35(±0.46)	0.76(±1.76)	0.490
Average BNP (pg/ml)	2184.38(±3406.08)	3379.62(±8204.52)	0.945
Peak BNP (pg/ml)	3164.31(±3444.76)	4335.62(±10860.48)	0.580
Admission BNP (pg/ml)	2095.37(±3284.15)	3427.63(±8389.59)	0.658
**Admission Vitals**	** **	** **	** **
Admission SBP (mmHg)	119.9(±47.51)	143.06(±35.87)	0.053
Admission DBP (mmHg)	58.5(±27.09)	80.66(±22.23)	**0.003**
Admission HR (bpm)	86.00(±19.56)	88.94(±24.99)	0.716
**Laboratory Data**			** **
Cortisol level (µg/ml)	36.73(18.82)	25.64(±16.38)	0.332
TSH (uIU/ml)	1.63(±0.44)	2.35(±2.91)	0.671
Free Thyroxine (ng/dl)	0.79(±0.07)	1.09(±0.43)	0.333
HDL level (mg/dl)	46.42(±18.34)	45.64(±14.81)	0.901
LDL level (mg/dl)	52.41(±17.81)	87.06(±34.34)	**0.016**
**Admission Laboratory Data**			** **
Admission hemoglobin (g/dl)	11.31(±2.08)	13.59(±1.87)	**0.001**
Admission sodium (mmol/L)	135.10(±3.93)	137.61(±3.24)	**0.020**
Admission BUN (mg/dl)	28.60(±16.59)	19.17(±10.51)	**0.009**
Admission creatinine (mg/dl)	1.65(±0.91)	1.07(±1.09)	0.101
Admission GFR	49.80(±28.44)	71.21(±27.04)	**0.016**
Admission lactic acid (mmol/L)	7.59(±5.18)	2.39(±2.11)	**0.001**
Admission albumin (g/dl)	3.22(±0.46)	3.89(±0.49)	**0.001**
**Average Laboratory Data**			** **
Average hemoglobin (g/dl)	10.45(±1.89)	12.52(±1.61)	**0.001**
Average sodium (mmol/L)	135.50(±4.48)	138.55(±2.60)	**0.001**
Average BUN (mg/dl)	37.65(±19.59)	17.69(±7.62)	**0.001**
Average creatinine (mg/dl)	2.01(±1.20)	0.97(±1.06)	**0.003**
Average GFR	54.50(±58.15)	80.60(±28.03)	**0.009**
Average lactic acid (mmol/L)	5.77(±0.87)	1.84(±0.93)	**0.001**
Average albumin (g/dl)	2.86(±0.59)	3.64(±0.55)	**0.001**

* Significant p values appear in **bold** font

** Brain natriuretic peptide (BNP), Systolic blood pressure (SBP), Diastolic blood pressure (DBP), Heart rate (HR), Millimeters of mercury (mmHg), Beats per minute (bpm), Thyroid stimulating hormone (TSH), High density lipoprotein (HDL), Low density lipoprotein (LDL), Blood urea nitrogen (BUN), Glomerular filtration rate (GFR)

Table 3 depicts the echocardiographic and cardiac catheterization data. Patients who died during hospitalization had a non-significant lower average EF of 36.5 (SD 16.5)% versus an average EF of 44.15 (SD 15.94)% in the non-mortality subgroup (p = 0.143). There was, however, a significantly higher degree of CAD in the mortality group versus the non-mortality group (p = 0.012).

**Table:** Table 3 Echocardiographic Data and Cardiac Catheterization Data for the Mortality and Non-Mortality Subgroups

**Echocardiographic Data**	**Mortality (n=10)**	**Non-Mortality (n=167)**	**p-value**
	**Mean (±SD)**	**Mean (±SD)**	
Ejection Fraction (%)	36.5(±16.5)	44.15(±15.94)	0.143
Diastolic Dysfunction	1.0(±1.05)	0.73(±0.82)	0.452
Aortic stenosis****	0.30(±0.95)	.13(±0.54)	0.347
Aortic regurgitation****	0.2(±0.54)	0.13(±0.39)	0.580
Mitral regurgitation****	1.0(±1.05)	0.73(±0.82)	0.326
RVSP (mmHg)	37.04(±8.83)	36.55(±11.66)	0.907
**Cardiac Catheterization Data**	** **	** **	** **
LVEDP (mmHg)	24.5(±9.99)	19.6(±6.54)	0.082
Degree of CAD*****	2.67(±1.32)	1.93(±0.83)	**0.012**

* Significant p values appear in **bold** font.

**** Right ventricular systolic pressure (RVSP), left ventricular end-diastolic pressure (LVEDP), Coronary artery disease (CAD).

### EF < 35% versus EF ≥ 35% Subgroups

Further data analysis was performed comparing EF <35% versus EF ≥ 35%. (Table 4) In patients with an EF < 35% the mortality rate was 8.2%. In patients with an EF < 35% the degree of CAD was higher (p = 0.011), Killip classification was also higher (p = 0.002), and LVEDP levels were higher (p = 0.005) than in patients with an EF ≥ 35%. There were also significant differences in admission brain natriuretic peptide (BNP) lab values (p = 0.009), peak BNP (p = 0.005), and average BNP levels (p = 0.003). Other admission laboratory data in patients with an EF <35% were significantly different than in patients with an EF ≥ 35% (Table 4).

**Table:** Table 4 Clinical Variables, Echocardiographic Data, Cardiac Biomarkers, Admission Laboratory Data, Laboratory Data for the Sub-Group Analysis EF < 35% versus EF ≥ 35%

	**EF < 35% (n = 49)**	**EF ≥ 35% (n = 128)**	**p-value**
	**Mean (±SD)**	**Mean (±SD)**	
LOS (days)	7.41(±5.36)	4.46(±7.17)	**0.010**
LVEDP (mmHg)	22.26(±7.34)	18.88(±6.28)	**0.005**
Degree of CAD	2.24(±1.00)	1.86(±0.79)	**0.011**
Killip classification	2.31(±1.19)	1.72(±1.06)	**0.002**
**Echocardiographic Data**			
Diastolic Dysfunction	0.91(±1.08)	0.76(±0.85)	0.505
Mitral regurgitation	1.13(±0.95)	0.61(±0.75)	**0.001**
RVSP (mmHg)	40.48(±11.72)	34.99(±11.05)	**0.012**
**Cardiac Biomarkers**			
Average Troponin (ng/ml)	2.93(±8.88)	1.91(±5.48)	0.371
Admission Troponin (ng/ml)	0.93(±2.47)	0.67(±1.33)	0.371
Average BNP (pg/ml)	6289.49(±11303.98)	1357.91(±3145.84)	**0.003**
Peak BNP (pg/ml)	7783.81(±14790.68)	1703.02(±4736.49)	**0.005**
Admission BNP (pg/ml)	5954.38(±10970.68)	1534.04(±4645.63)	**0.009**
**Admission Laboratory Data**			
Admission hemoglobin (g/dl)	12.61(±2.33)	13.79(±1.67)	**0.001**
Admission sodium (mmol/L)	136.59(±4.36)	137.81(±2.76)	**0.029**
Admission BUN (mg/dl)	22.61(±14.08)	18.56(±9.55)	**0.030**
Admission creatinine (mg/dl)	1.42(±1.92)	0.98(±0.43)	**0.014**
Admission GFR	63.84(±29.71)	72.37(±26.33)	**0.065**
Admission albumin (g/dl)	3.71(±0.51)	3.91(±0.49)	**0.022**
**Laboratory Data**			
Average hemoglobin (g/dl)	11.59(±1.91)	12.71(±1.49)	**0.001**
Average sodium (mmol/L)	137.79(±3.65)	138.60(±2.40)	0.088
Average BUN (mg/dl)	22.04(±13.01)	17.59(±7.95)	**0.006**
Average creatinine (mg/dl)	1.34(±1.92)	0.91(±0.47)	**0.020**
Average albumin (g/dl)	3.31(±0.58)	3.71(±0.54)	**0.001**

* Significant p values appear in **bold** font.

** Length of stay (LOS), Left ventricular end diastolic pressure (LVEDP), Coronary artery disease (CAD), left ventricular outflow tract (LVOT), Right ventricular systolic pressure (RVSP), Brain natriuretic peptide (BNP), Glomerular filtration rate (GFR), Blood urea nitrogen (BUN),

### Univariate Logistic Regression

Variables that achieved statistical significance (i.e., p values < 0.05) from the univariate analysis were considered for inclusion in the non-parametric logistic regression model for in-hospital mortality. Variables that achieved significance from the model are found in Table 5. The Hosmer-Lemeshow goodness of fit test was used to ensure the model appropriately fit the data.^28^ As seen in Table 5, the p values calculated from variations in each of the admission labs of were found to be statistically significant predictors of hospitalization mortality (p values ranging from 0.028 to 0.001).

**Table:** Table 5 Univariate Logistic Regression for the Prediction of Patients with Mortality in Takotsubo Syndrome

**Variable**	**Coefficient (*B*)**	**Wald X^2^**	Sig.*	**Odds Ratio**	**95.0% Confidence Interval**	
					**Lower**	**Upper**
Admission Diastolic BP	-0.053	0.018	**0.003**	0.948	0.915	0.983
Admission Hemoglobin	-0.48	10.011	**0.002**	0.619	0.46	0.833
Admission Hematocrit	-0.147	8.272	**0.004**	0.863	0.781	0.954
Admission Sodium	-0.174	4.937	**0.026**	0.841	0.72	0.98
Admission BUN	0.049	5.787	**0.016**	1.05	1.009	1.093
Admission GFR	-0.034	5.676	**0.017**	0.966	0.939	0.994
Admission Lactic Acid	0.444	4.828	**0.028**	1.559	1.049	2.318
Admission Albumin	-2.72	12.544	**0.001**	0.066	0.015	0.297

* Significant p values appear in **bold** font.

### Cox Proportional Hazards Regression

When comparing the mortality and non-mortality subgroups, the statistically significant variables from univariate logistic regression (Table 5) were considered for analysis in a multivariate cox regression model. Ultimately, admission hemoglobin, admission creatinine, admission systolic blood pressure, and admission albumin were included in the final Cox regression model. Of these variables, admission hemoglobin was significant with a hazard ratio of 0.760 (95% CI: 0.594-0.972, p = 0.029). Thus, for each unit decrease in admission hemoglobin in TTS patients there was an associated increase in in-hospital mortality by 24%

### Multivariate Linear Regression

A simple linear regression was calculated to predict differences between the EF <35% and EF ≥ 35% subgroups of sample patients. The statistically significant variables that were considered were taken from the univariate logistic regression results and entered into a multivariate linear regression model. Ultimately, degree of CAD, MR, admission hemoglobin, and admission creatinine were the variables used in the model. A significant regression equation was found F(4, 7.189) = 4.813, p < 0.001, with an R^2^ of 0.151. Although not depicted in a separate table degree of CAD (B = -0.166, p = 0.024), mitral regurgitation (B = -0.199, p = 0.009), and admission hemoglobin (B = 0.169, p = 0.025) were shown to be significant predictors of having an EF <35%.

## DISCUSSION

TTS is a transient heart failure syndrome that can typically present with complications at similar rates to ACS patients in hospital settings.^6–8,24^ The in-hospital TTS mortality rate in this study was 5.65% which was similar to previous studies.^5,6,8,9,11,12^

The presence of co-morbid conditions has also been associated with poor outcomes in TTS.^7,29–31^ In their 2010 article, Brinjikji et al, reported renal impairment as a critical predictive factor related to TTS-related mortality.^29^ Consistent with this 2010 study, our sample patients with ESRD also had a significantly higher mortality rate. Endothelial dysfunction, oxidant stress, vascular calcifications, and inflammation from renal dysfunction also likely contribute to worse TTS patient outcomes.^32^

Previous studies have demonstrated a similarly higher incidence of cancer in TTS compared to the general population.^33,34^ In one 2016 study, 28.5% of TTS patients were diagnosed with cancer, and the presence of cancer was found to be an independent predictor of cardiac and all-cause death in TTS.^35^ Although this pathologic mechanism is not well understood, it has been postulated that possessing a malignancy may enhance patients’ neurohormonal activation and the inflammatory response during the acute phase of TTS.^35^

In our study, degree of CAD was significantly associated with higher degree of cardiomyopathy and in-hospital mortality. The Mayo Clinic criteria indicate that the absence of obstructive CAD is still possible with a diagnosis of TTS, and previous studies have reported non-CAD levels in up to 19% of TTS patients.^5,18,36,37^ Modern techniques such as fractional flow reserve and intravascular ultrasound have now provided objective measurements to evaluate potential obstructive lesions to help delineate TTS from obstructive CAD. Parodi et al (2013) stated that TTS and CAD were not mutually exclusive and up to 10% of TTS patients may have been missed due to their exclusion of patients with documented CAD.^37^

It remains especially important to recognize the presence of CAD in TTS as it is associated with poor outcomes. In Bill et al. (2017), CAD in TTS patients had significantly lower EF, higher risk of cardiogenic shock was an independent predictor of mortality when compared to non-CAD TTS patients.^36^ In 2016, the European Society of Cardiology recognized CAD as a risk factor for more severe heart failure during acute TTS episodes.^2^

Lower hemoglobin levels place an extra demand on cardiac output and decrease blood viscosity (i.e., thickness), leading to vascular (arterial and venous) dilation, which in turn leads to increased preload.^38^ In a chronic anemic state, LV hypertrophy can develop and ultimately lead to LV dilation and heart failure.^30^ Jankoswka et al, (2014) found iron deficiency is associated with higher mortality in a 12-month follow-up of heart failure patients.^32,39,40^

In our study, there was no significant difference found in BNP levels between the mortality and non-mortality sample subgroups. However, low BNP levels have been connected with favorable prognoses elsewhere.^8,41^ However, the correlation of BNP levels with hemodynamic parameters such as LVEDP are not as reliable in TTS.^2,7,42^ Our study found that patients with an EF < 35% had significantly higher elevations of BNP and higher LVEDP values.

The mechanism of TTS is not entirely understood, although it is believed to be from a surge of catecholamines causing vascular dysfunction and direct myocardial stunning. Initial surges of catecholamines appear to correlate with NT-proBNP levels and the extent of LV systolic function.^43,44^ Since epinephrine and norepinephrine work on the beta receptors in the ventricular myocardium, it would appear beta blockers would be the ideal treatment of this syndrome in acute TTS.^13^ However, patients receiving beta blockers in earlier studies have not demonstrated a significant difference in 30-day mortality cardiovascular complications, or TTS recurrence.^17,18^

Inflammation also likely plays a significant role in the pathogenesis of TTS.^35^ Previous studies have suggested a benefit in ACEI and ARBs via the renin-angiotensin-aldosterone pathway and direct anti-inflammatory properties on the myocardium.^18^ The authors found no prospective studies to date examining their use in TTS.

### Study Limitations

We should acknowledge several study limitations. This was a single-center study with predominantly Caucasian population so our results may not be generalizable to other ethnic groups. This study was limited by its retrospective design and the sample subgroups may have differed in unmeasured ways. None of our sample patients underwent cardiac magnetic resonance imaging to differentiate between acute infarct or myocarditis as the etiology of their cardiomyopathy.^2^

Finally, due to our low number of mortality cases (n = 10) our regression model may have contained more parameters that could be justified by the data. However, we included only minimal parameters found to be significant through univariate logistic regression to reduce our risk of using an over fitted predictive model. Finally, we did not examine right ventricular involvement in these cases which has been associated with a poor prognosis in TTS.^45^

## CONCLUSIONS

In conclusion, these results demonstrate that patients who present with TTS on admission with hypotension, anemia, low albumin levels, elevated lactic acid and renal dysfunction appear to be at higher risk for in-hospital mortality. In addition, anemia may comprise one of the strongest predators of in-hospital mortality.

The lack of evidence-based therapies for this condition highlights the ongoing need for studies identifying those TTS patients at higher risk for complications. Further research is warranted to determine the most effective therapies for TTS patients so frequently already prone to health complications. Cardiology providers require further evidence from larger prospective research samples to identify optimal treatment approaches for these higher risk cardiology patients.

### DISCLOSURES

The overall results of this study were presented as a poster presentation at the Statewide Campus System Poster Day, May 2017. This study was supported by the Genesys Regional Medical Center Department of Academic and Clinical Research.

### Conflict of Interest

The authors declare no conflict of interest.
